# Analyzing the impact of COVID-19 on the electricity demand in Austin, TX using an ensemble-model based counterfactual and 400,000 smart meters

**DOI:** 10.1007/s43762-023-00095-w

**Published:** 2023-05-06

**Authors:** Ting-Yu Dai, Praveen Radhakrishnan, Kingsley Nweye, Robert Estrada, Dev Niyogi, Zoltan Nagy

**Affiliations:** Department of Civil, Environmental and Architectural Engineering, The University of Texas at Austin, Austin, 78712-1700 Texas USA

**Keywords:** COVID-19, Machine learning, Data synthesizer, Socioeconomic factors, Building energy performance simulation, Ensemble model

## Abstract

The COVID-19 pandemic caused lifestyle changes and has led to the new electricity demand patterns in the presence of non-pharmaceutical interventions such as work-from-home policy and lockdown. Quantifying the effect on electricity demand is critical for future electricity market planning yet challenging in the context of limited smart metered buildings, which leads to limited understanding of the temporal and spatial variations in building energy use. This study uses a large scale private smart meter electricity demand data from the City of Austin, combined with publicly available environmental data, and develops an ensemble regression model for long term daily electricity demand prediction. Using 15-min resolution data from over 400,000 smart meters from 2018 to 2020 aggregated by building type and zip code, our proposed model precisely formalizes the counterfactual universe in the *without COVID-19* scenario. The model is used to understand building electricity demand changes during the pandemic and to identify relationships between such changes and socioeconomic patterns. Results indicate the increase in residential usage , demonstrating the spatial redistribution of energy consumption during the work-from-home period. Our experiments demonstrate the effectiveness of our proposed framework by assessing multiple socioeconomic impacts with the comparison between the counterfactual universe and observations.

## Introduction

In March 2020, the World Health Organization declared COVID-19 to be a global pandemic. To suppress the infection, governments enacted a number of social distancing policies (SDPs), such as lockdown, social distancing recommendations, and work-from-home orders. Such measures have led to a socioeconomic shock to the global systems. The environmental and climate changes were notable  healthcare facilities also experienced challenge (Kaye et al., 2021), international supply chain were disrupteds (Inoue & Todo, 2020), affecting global economy (Shan et al., 2021), and tourism (Bureau, 2020).

Interlinked to these various socioeconomic changes, industrial energy and electricity systems experienced a considerable shift in demand, largely due to the partial or full closure of industrial activities. A global decline of 5-6% is expected in both energy and electricity demand in developed countries, such as the US (9%), with largest reduction expected in the European countries (11%) (International Energy Agency (IEA), 2020). On the other hand, residential energy demand is expected to rise due to increase in home stay, remote working, online shopping, powering home appliances, and heating or cooling homes (Birol, 2020). The increase in the energy bills for residential consumers, could further lead to some social vulnerabilities. 

To better decompose the pattern changes in energy consumption, we roughly define our analysis duration into four periods to manifest the different situation in Austin which shown in Table 1. Each period represents a unique stage while epidemic evolves by time. First period points to the moment when the COVID-19 just officially outbroke in the state, and second period states the implementation of opening policies from the Texas government to abate the restriction of business and industrial behaviors. Third period marks the intensifying COVID cases after the open phases from July to August while the final period indicates the steady growth of COVID cases after the recovery from the previous wave.

**Table 1:** Four stages definition in Austin of year 2020

Definition	Period
Pandemic outbreak	Mar. 29 - Apr. 30
Texas Open Phase	May 1 - Jun. 30
Upsurge in COVID cases in Austin	Jul. 1 - Aug. 31
The steady increasing cases phase	Sep. 1 - Dec. 31

The aim of this study is twofold. First, to develop a computational model to estimate the energy demand in Austin, TX as if there was no COVID, i.e., a counterfactual synthesizer for electricity demand. We are in particular interested in residential electricity demand, aggregated by zip code. Second, using the developed model, we estimate the impact of COVID by comparing our model’s output at different spatio-temporal scales with smart meter data. We inspect the relationship between energy consumption differences and social variables such as poverty, income level, and race data through different periods.

## Related work

### COVID-19 and building energy demand

Since the pandemic began, lots of researchers have started to explore the effect of COVID-19 on electricity demand in different places such as universities, residential buildings, and even at neighborhood level (Berg et al., 2022; Abu-Rayash & Dincer, 2020; Gaspar et al., 2021; Abdeen et al., 2021; Abulibdeh, 2021; Bielecki et al., 2021; Chihib et al., 2021; García et al., 2021; Bahmanyar et al., 2020). Berg et al. (2022) aim to find the electricity consumption changes due to COVID-19 on single-family houses on rural Iowa. They find that 54 percent of buildings had a significant change in their non-weather-related consumption in 2020 compared to their previous years. Abu-Rayash & Dincer (2020) have done the analysis for Ontario, Canada for the month of April and found that overall, the electric demand for the province of Ontario is reduced notably and daily demand reductions were observed on weekends. The results from Gaspar et al. (2021) show that for university buildings energy consumption fell by 19 percent during the post-pandemic year. They revealed that energy consumption variation was higher in libraries followed by teaching buildings. The analysis by Abdeen et al. (2021) indicate that the imposed lockdown resulted in increasing residential demand by 11-20 percentage for 500 homes in city of Ottawa Canada. Abulibdeh (2021) investigates the impact of the pandemic on the spatial patterns of electricity consumption in six socioeconomic sectors (residential (villa and flat), industrial, commercial, government, and productive farms) in the State of Qatar and concluded that industrial and commercial sectors were the most affected by the pandemic. Bielecki et al. (2021) too observe increase in daily electricity demand is observed with practically unchanged peak loads in region of Poland. Chihib et al. (2021) aims to measure the impact of closing the campus on the energy use of its different facilities and find that the situation of closing the campus facilities during the COVID-19 outbreak influenced the overall energy consumption of the campus. However, the impact magnitude varies from one category to another. The research category is the least influenced by the outbreak situation and library building is the most influenced. García et al. (2021) results show that residential customers have increased their consumption by around 15 percent during full lockdown and 7.5 percent during the reopening period. In contrast, globally, non-residential customers have decreased their consumption by 38 percent during full lockdown and 14.5 percent during the reopening period. Bahmanyar et al. (2020) compares the impact of different containment measures taken by European countries in response to COVID-19 on their electricity consumption profiles and concludes that Spain, Italy, Belgium, and the UK with severe restrictions, the weekday consumption iss considerably reduced and energy consumption profiles are similar to pre-Pandemic weekend profiles for the same period in 2019. However, for countries with less restrictive measures, the decrease in power consumption was lower.

### Socioeconomic status with energy consumption

Socioeconomic status is also a critical factor in energy consumption. Various areas will affect the business and residential behaviors which lead to the diverse patterns for different zip code areas (Harputlugil & de Wilde, 2021; Fu & Zhai, 2021). The review indicates that most of the relevant researches focus on the technical side instead of social issues. Moreover, most of the study fields are at individual building scales due to the lack of a comprehensive dataset. Robinson et al. (2019) argue that spatial variety of social vulnerability in households that the assessment of energy poverty should be determined by a geographically weighted index. Elnakat et al. (2016) have done the zip code level research for the correlation between the socioeconomic distribution and energy consumption in San Antonio, Texas. Gender, age, and income level have been linked to the dynamic influence of energy utilization. High energy consumption communities are able to link with higher levels of education, income, owner-occupied percentage while the population density is on the contrast side. Prol & Sungmin (2020) demonstrate the overall decreasing energy consumption during COVID-19 in country scale. They also state the nonlinear relationship between policy stringency and daily recession in electricity usage. Developing a nonlinear model to address the interrelation between energy consumption and social factors is urgent. However, few studies tackle the social issues in the energy domain with a nonlinear relationship driven by machine learning models. Those are being said by Harputlugil & de Wilde (2021), either too focused on the technical parts or lack of the data on evaluating stage.

### Statistical methods in energy consumption

Methods for predicting energy consumption can be categorized into two types: physical models and statistical models. This study applies statistical methods due to its light demand for computational resources and its re-productivity over each smart meter data. There are several papers using linear methods like Berg et al. (2022); Abdeen et al. (2021). However, those methods require equations that are defined by researchers. The manually defined relation are not necessarily suitable for all types of energy data. In addition, it is a time-consuming process. Finer resolution smart meter data also leads to the inadequacy of explaining energy consumption data with merely linear models. In recent years, due to the enhancement of machine learning techniques, there are also some studies that applied machine learning techniques to analyze energy consumption (Abu-Rayash & Dincer, 2020; Olu-Ajayi et al., 2022; Robinson et al., 2017). Abu-Rayash & Dincer (2020) apply k-means clustering to identify consumption behaviors while Olu-Ajayi et al. (2022) investigate multiple machine learning methods to predict the building energy data. In the comparison by Olu-Ajayi et al. (2022), they state the superior performances from machine learning methods. Moreover, the same suggestion is shown up in Robinson et al. (2017)’s study states that XGBoost surpassed the linear regression in their study. Still, there are few applications that apply a large dataset with more than thousands of instances with a sub-hourly scale to understand energy consumption.

### Research aims

This research plans to quantify the impact of COVID-19 on a fine scale. Particularly, this study targets to identify the non-linearity of energy consumption based on 400,000 smart meters using an ML-based data synthesizer in Austin. Therefore, the aims of our work are:

1. Utilizing a large dataset with more than 400,000 smart meters to quantify the impact of COVID-19.
2. Developing multiple machine learning models to synthesize the energy demand data in a “without COVID-19” scenario.
3. Establishing an ensemble method to combine the output from multiple models and a detailed comparison between different weighting schemes.
4. Conducting an in-depth city-scale analysis for energy demand variation due to the impact of COVID-19.

## Methodology

**Fig. 1:**
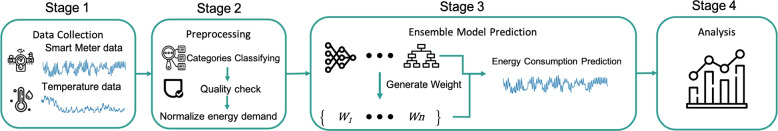
Overall flowchart for the methodology

The overall flowchart of the methodology to analyze disaster-based impact on energy consumption is shown in Fig. 1. The procedure starts with the data collection including environmental data and smart meter data. Then, the smart meter data is regrouped to reduce the initially large number of customer types. The following preprocessing procedures include the quality check that examines the integrity of the smart meter data and the normalization based on the meter used at each target time step. Then, the ensemble model is developed to investigate the difference between the energy consumption in pre-COVID and during-COVID period. Finally, the impact of COVID-19 on the energy demand is analyzed.

### Preprocessing

#### Building type aggregation

Due to the large number of customer data types, we reorganize the smart meter data into residential, commercial, and other types based on their building types. We use correlation to validate the unitarity of the used building types as the criterion of the regrouping. For each smart meter data, the Pearson correlation coefficient with other data are calculated and aggregated by their metadata like zip code and building type. After investigation, the correlation matrix of building type level is generally higher than the others (individual and aggregated at zip code level). The result of the correlation analysis indicates that the building type is highly related to energy consumption patterns. For instance, among all the commercial building types, restaurants have similar patterns with the fast food restaurants, and convenience stores are also alike in their electricity usage patterns. Also, the residential buildings are often associated such as the type “GARAGE APARTMENT” related to the type “MULTI FAMILY”, indicating the similarity between the energy usage data of close building types. Based on the result of this pre-analysis, this aggregation is included in the proposed framework to accelerate the analysis.

#### Quality check

To match the temporal resolution of the environmental data and improve the efficiency of the whole process, the raw 15-minute resolution energy consumption data are resampled to coarser time scales such as hourly, daily, and weekly. Based on the target time frequency, a preliminary quality scrutinization is applied to check the data. The checking method is that the data would be approved if the number of data within the desired frequency is more than half of the total number. After that, we trim the extreme values from the data which are 5% data from top and bottom respectively.

#### Normalization

The normalized energy consumption data $E_{ijX}$ is computed as

1 $$\begin{aligned} E_{ijX} = \sum _{B=1}^{C} \left[ \left( \frac{1}{N_{ij}} \sum _{T=i}^{N_{ij}} E_{T}\right) \times \frac{M_{B}}{\sum _{B=1}^{C} M_{B} } \right] \end{aligned}$$

where *i*, *j*, and *X* are the start, end time step, and the target category, respectively, and *C* is the number of data in the category. $N_{ij}$ is the number of data from *i* to *j*, and $E_T$ is the energy consumption at the target moment *T* which starts from *i* to *j*. *M* denotes the area of the building.

#### Feature design

Three features are used to model energy consumption, time components, and outdoor temperature. Time features are generated as the sine and cosine waves of a day, sine and cosine waves of a year, day of year, and month. The outdoor temperature is extracted from the weather station including in Integrated Surface Database (ISD) of National Centers For Environmental Information, NOAA at Camp Marby, Texas.

### Ensemble model prediction

In practice, several regression models are available to estimate energy consumption, but none of them is perfectly accurate and each method may be making mistakes in different facets. Thus, stacking multiple different regression methods may lead to performance improvement over individual models. Multi-model ensemble is a method in which the predictions of a collection of models are weighted averaged. In our study, Random Forest(RF) (Ho, 1995), XGBoost (Chen & Guestrin, 2016), AdaBoost (Adaptive Boosting) (Freund & Schapire, 1997), Histogram-Based Gradient Boosting Regressor(HGBR), and LightGBM (Ke et al., 2017) are deployed as the predictor in this case. Five voting regressors are selected through comparative tests. The neural network was originally considered in the experimental phase, but since this approach created a much smoother result than the others, we excluded this method in this study to avoid overfitted prediction.

In this work, the ensemble learning model mainly includes two stages. The first stage is to use 10-fold cross-validation with training data. The algorithm with better accuracy and operation performance is selected to be voted. According to the training accuracy of each algorithm, the dynamic weight of it are set. For the weighting scheme, the formula is presented as,

2 $$\begin{aligned} W = \left\{ \begin{array}{ll} \frac{n}{\sum _{i=1}^{n} \mid x_{i} - x\mid }, &{} \text {if scheme = 1/MAE}\\ \sqrt{\frac{n}{\sum _{i=1}^{n} (x_{i} - x)^2}}, &{} \text {if scheme = 1/RMSE}\\ \frac{n}{\sum _{i=1}^{n} \mid \frac{x_{i} - x}{x}\mid }, &{}\text {if scheme = 1/MAPE} \end{array}\right. \end{aligned}$$

where *n* is the number of validation data, and $x_{i}$ and *x* are the prediction and observation of the energy consumption. The weight defines the preliminary confidential index of each method. The second stage is to output the preliminary prediction result from each regressor, and then calculate the final result using the voting algorithm. The generated result will be compared with the observation to see the impact of the disaster.

To evaluate the effectiveness of the proposed method, mean absolute error (*MAE*), root mean squared error (*RMSE*), and mean absolute percentage error (*MAPE*) are calculated. Using MAE as one of the evaluation metrics is to see the overall differences between the observations and the predictions, and using MAPE is to investigate the percentage difference, which reflects the differences in a comparable way, since the number is divided by the observation. RMSE is to inspect the outliers of the prediction so that we could see the degree of generating unreasonable prediction by that index. To further confirm that the ensemble model could properly reproduce the energy consumption through the training data, we first compare the performance between each method including in the ensemble model using 10-fold cross-validation. Then, three different weighting schemes are presented to see the accuracy of stacking the prediction from single estimators. Finally, the ablation test is implemented to demonstrate the stability of the ensemble mode.

### Analysis on the impact of COVID-19

To investigate the impact of COVID-19, the ensemble model described in the previous section is applied to generate the synthesized energy consumption using input data like time features i.e. day of year, hour of day, and seasonality and the air temperature in this study. The experiment setup uses the period before COVID-19, Years 2018 and 2019, as the training data, so that the prediction from the model is treated as the counterfactual output in the “without COVID-19” scenario in the future. Therefore, to quantify the difference between the periods of COVID and pre-COVID, the observation in 2020 and the counterfactual prediction in 2020 using the ensemble model are compared in Section 4.

In this study, total 402785 smart meters data in Austin area are collected by the City of Austin with 43 building types, covering 46 zip code areas, under 5 different counting measurements. The period in the dataset mostly covers from 1, Jan. 2017 to 1, Oct. 2021 while the raw temporal resolution is 15 minutes. However, due to the aim of analyzing the long-term impact of COVID-19, we examine the data on different temporal resolution which are hourly, daily, and weekly. The date of Texas government policies response are referred from Intelligent Environments Laboratory and Environmental Engineering (2021) while the demographic and economic data of Austin are referred from Austin (2021).

To further analyze the social impact of COVID-19, the Social Vulnerability Index (SVI) provided by Centers for Disease Control and Prevention (CDC) is utilized to investigate the relationship between socioeconomic status and building energy consumption. The CDC SVI dataset is collected in 2018 and indicates the vulnerability status of every U.S. Census Tract. To ensure the SVI data is on the same geometric scale, we applied Crosswalk Files released by Policy Development and Research (PD &R) from The U.S. Department of Housing and Urban Development’s (HUD’s) Office. Crosswalk Files are derived from data in the quarterly USPS Vacancy Data and are highly responsive since the data updates quarterly. By using the residential ratio projected to each zip code level, we transferred the SVI index and race data from the scale of the census tract into the zip code level. Moreover, the race data by U.S. Census Bureau is also implemented in this study to demonstrate the correlation with energy consumption. We compare two kinds of race data: One is to separate the single race including White alone, Black or African American alone, American Indian and Alaska Native alone, Asian alone, and Native Hawaiian and Other Pacific Islander alone, and the other one is Hispanic or Latino.

On the other hand, the percentage of energy consumption differences based on each zip code is determined based on the difference between the output of the proposed model and real observations during the COVID period. We also consider the raw difference in energy consumption, but since it will make the difference too huge to observe the insight of different social variables, this study use percentage form to conduct the analysis. We then establish the linear relationship between social variables and the percentage of energy consumption to see if solid dependencies are aligned. However,

## Results

### Model validation

Table 2 summarizes the performance between Ridge linear regression, XGBoost, and our ensemble methods. The table demonstrates the improvement compared to the linear methods, and also for single nonlinear ML methods like XGBoost.

**Table 2:** MAE, RMSE, and MAPE differences of different ML methods

Evaluation Metrics	Ridge	XGBoost	Ensemble (ours)
Commercial Type			
MAE	124.61	118.28	**115.22**
RMSE	151.01	136.94	**132.97**
MAPE	19.26	18.27	**18.11**
Residential Type			
MAE	251.46	70.94	**63.53**
RMSE	300.60	97.85	**83.99**
MAPE	16.20	4.50	**4.08**

**Table 3:** Weighting Scheme comparison for MAE, RMSE & MAPE

Weighting Strategy	1 / MAE	1 / RMSE	1 / MAPE	Average
Commercial Type				
MAE	117.85	119.48	**117.47**	118.59
RMSE	139.62	**135.11**	142.21	142.65
MAPE	19.28	18.42	**18.01**	0.18.91
Residential Type				
MAE	65.83	**63.86**	66.85	65.76
RMSE	90.06	85.66	**84.25**	92.39
MAPE	4.21	**4.11**	4.31	4.47

**Table 4:** Cross Validation comparison for single Method using MAE, RMSE & MAPE

Evaluation Metrics	AdaBoost	Random Forest	HGBR	XGBoost	LightGBM
Commercial Type					
MAE	**112.67**	117.82	126.06	132.48	125.34
RMSE	**129.27**	139.73	147.59	158.54	149.46
MAPE	**17.47**	18.47	19.84	20.72	19.72
Residential Type					
MAE	102.47	**64.19**	68.37	68.60	68.95
RMSE	126.08	**89.80**	95.76	92.24	92.17
MAPE	7.22	**4.10**	4.39	4.42	4.42

#### Weighting scheme test

There are multiple weighting methods to merge the result for the estimators, and inspired by Merrifield et al. (2020), this study applies the variant of RMSE distance weighting, that support the scaling based on the MAE, RMSE, and MAPE metrics. They conclude the advantage of the RMSE-based independence scaling, which include allowing for degrees of dependence. Therefore, in this section, a simple comparison that uses different metrics as the weighting schemes are summarized.

Table 3 shows the result using different strategies to generate the weight for the ensemble model. Overall, we could observe that both commercial and residential types of energy consumption are better estimated by RMSE and MAPE schemes. They achieve the best performances compared to using MAE or just averaging the result equally. Although using MAE as weighting can achieve the nearly lowest MAE values, the performances of other two metrics are not as precise as the RMSE and MAPE ones. Besides different strategies, we could also conclude the stability of the ensemble model. The metrics of all four schemes are close and comparable with each other, which indicates the relatively high stability to those single models in Table 4. MAPE scaling scheme is deployed to the commercial data while the RMSE scaling scheme is deployed to the residential data in the later sessions.

#### Ablation study on each algorithm

To further validate the stability of the ensemble model, the ablation test is also implemented. In the experiment, we trim one method out at a time to compare the performance without that predictor, and the result is shown in Table 5. We could find consistency in the result of Table 4 and 5 that commercial data is more depend on the prediction of AdaBoost with the proof of largest values on all the metrics. However, the logic is not the same as the result of residential energy consumption. The performances without each method are close, and for RMSE, it is surprising that the one without AdaBoost has largest value since there is a gap with the poor result of AdaBoost in Table 4 with other methods. That also confirms the stability of the ensemble method and the predictability of residential energy usage.

**Table 5:** Ablation Comparison for each method

Methods Without	AdaBoost	Random Forest	HGBR	XGBoost	LightGBM
Commercial Type					
MAE	122.09	121.26	116.72	116.98	117.26
RMSE	147.41	141.87	139.29	136.76	142.72
MAPE	19.99	19.13	18.01	18.54	18.82
Residential Type					
MAE	66.08	67.60	63.04	68.27	63.57
RMSE	91.07	88.39	89.78	88.64	83.71
MAPE	4.21	4.31	4.31	4.51	4.17

### Counterfactual model prediction

#### Changes during social distancing period

**Fig. 2:**
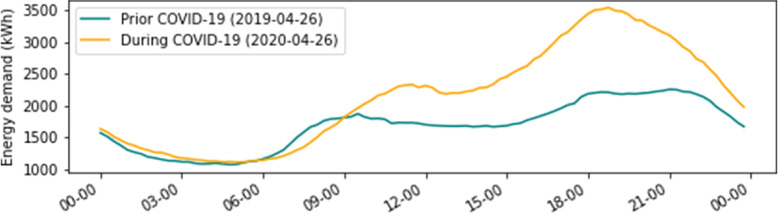
Time series comparison during the period prior COVID-19 and during COVID-19 in residential buildings

A preliminary visualization between the period prior to and during COVID-19 is shown in Fig. 2. We can see that the energy demand in different periods has unique patterns during the same day. In commercial buildings, the demands in the prior period are always higher than the data during the pandemic, and the variation between daytime and night time is also higher. That is consistent with the fact that after the implementation of SDPs, most of the commercial activities are forced to stop. For the residential building, the overall difference is not that large, but the patterns are slightly different during the working hours (8:00 - 17:00), there is more energy consumption during the COVID-19 period, which matches the working from home policies.

**Fig. 3:**
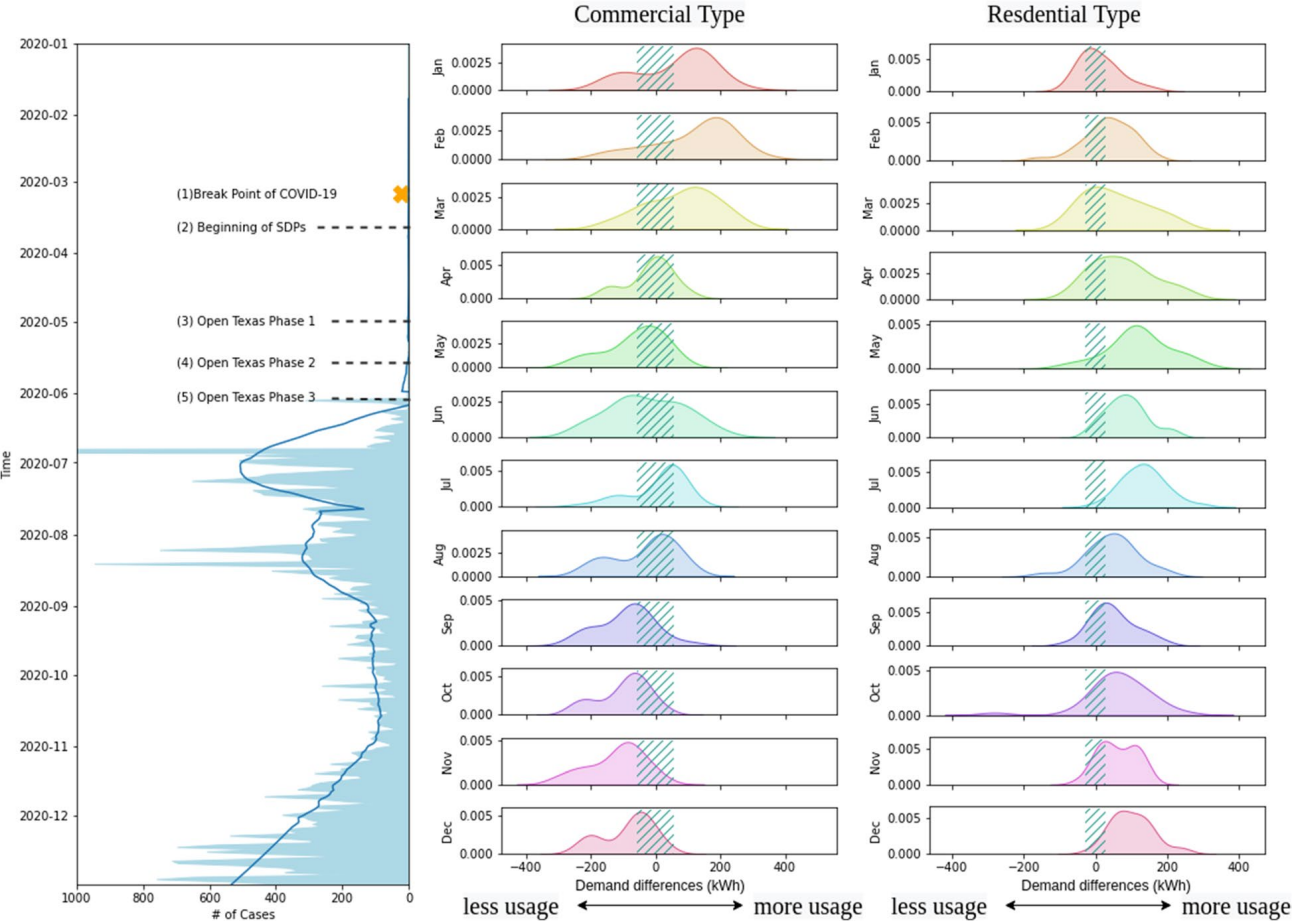
Comparison of energy consumption patterns - prior and during the SDPs

In order to further investigate the impact that has been brought by the pandemic, the monthly comparisons between the counterfactual predictions and the observations are shown in Fig. 3. The way of visualization is using the observations to subtract the predictions from the ensemble model, and each distribution plot represents the aggregation of differences. This figure demonstrates the impact of COVID-19 since March 2020. The peaks of commercial distributions start to shift since the breakpoint of COVID-19 (stage (1) in Fig. 3), which is moving from more usage than before to less usage until June. Also, the residential distributions are affected to move from the middle of the range of over usage during the same period. The reason is the implementation of SDPs. Due to the work-from-home, lockdowns, or even the social distancing recommendation, people change their patterns of life to avoid the pandemic. Those policies such as staying home for work directly enlarge the energy consumption of condos, townhouses, and single family building types, and the restaurant or offices are decreased during the daytime by the same basis.

Starting from May 1, 2020, Texas Governor Greg Abbott announces the state may begin the first phase of the three-phase reopening plan in Texas from SDPs as the stage (3) in Fig. 3. Retailers, restaurants, movie theaters, museums and libraries, contact-free outdoor sports, places of worship, and single-person offices, nearly all of which are subject to a 25% occupancy restriction are reopened since the first phase. Face coverings are only recommended in Texas and no state or local official can impose a civil or criminal penalty for failure to wear a face covering. In the regard proposed by The Governor’s Report to Open Texas, the state’s chief medical officer’s portion of the report requests, among other things, continued social distancing, limited physical contact, and use of face coverings and, in support of continued telecommuting, advises individuals to “stay home if you can.” Those three phases gradually fix the deviation of the distribution brought from the impact from COVID-19 in Fig. 3. During May and June, the residential distributions progressively shift back to the middle, and the commercial distributions are moving to the right like the estimations in February, hinting back to the patterns prior to the pandemic. Those findings illustrate the fact that citizens in Austin are following the policies to re-operate their lives back to the pattern before COVID-19.

However, COVID-19 had not been ended there. Another peak that we could identify from the COVID cases plot along the time in Fig. 3 is July. Austin was hit by another wave in July while recovering, and this made the patterns of energy consumption fluctuate again. The distribution is moving back to what it was prior to the pandemic, and related to the peak cases number, the distribution starts to change again. The commercial energy consumption data is mostly predicted in the range of validation, but starting from August, the impact of COVID makes that shift to less energy usage again, which forms a new usage pattern gradually. Relatively, the residential data suddenly deviates from the trend of moving less than pre-COVID period. Those observations conclude the high correlation with the COVID-19 cases.

During the latter months of 2020, the daily positive cases of COVID-19 just stayed at a certain level constantly compared to the first half of 2020. A permanent change in the data is formed. Citizens in Austin seem to be more accustomed to the adaptive lifestyle for COVID. The motion starting from July in the commercial distribution finally ended at a stage that used less electricity in most of the time. On the other side, the result comparing the counterfactual universe and observations demonstrates that demanding more energy from residential buildings during December. The dynamics in both building types all conclude the fact that COVID-19 has changed how people live in Austin in a permanent way.

#### Linked to the social: income and race

**Fig. 4:**
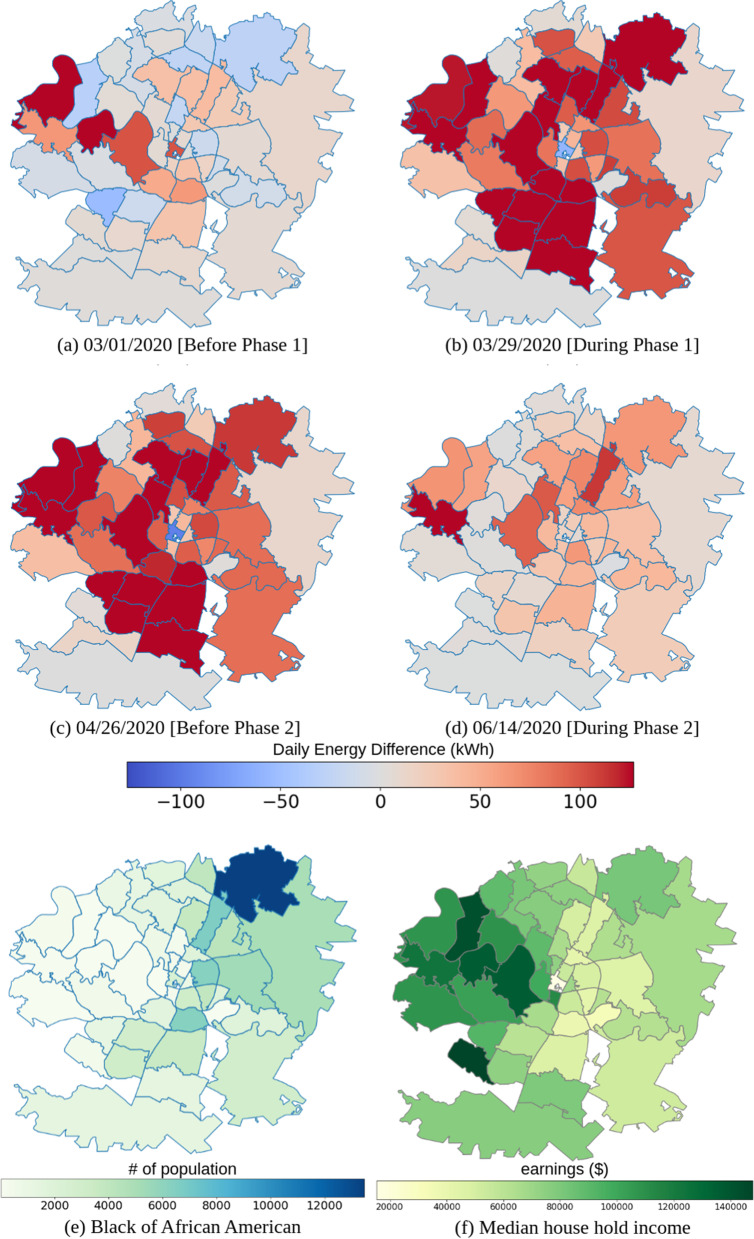
Spatial comparison of residential energy consumption patterns for (**a**) before implementations of SDPs, (**b**) after implementations of SDPs, (**c**) before Texas Reopening Phases, and (**d**) after Texas Reopening Phases in 2020, (**e**) Demographic distribution of Black or African American, and (**f**) Economic distribution for median household income

Decomposing the socioeconomic impact of COVID-19 in Austin is illustrated in this section. Figure 4 depicts the difference between the counterfactual universe, which is assumed to be the energy usage without the pandemic, and the observation which is the actual energy usage during the pandemic. The red area means that more usage is detected while the blue area is less. The socioeconomic references are presented in Fig. 4(e, f) based on the data from Austin (2021), including the race distribution and the median income level for the whole Austin area in zip code level. The residential consumption is uniform in that almost the same color covers the whole Austin in Fig. 4. The result indicates a huge over usage of the residential type since the implementation of SDPs by comparing Fig. 4(a) and (b). The statistics especially depict the focus on the west side and north side of Austin, and that can be related to Fig. 4(e, f) where people with higher income live. That relation between income and residential building basically implies that people with higher incomes increased their electricity demand to adapt the pandemic.

**Fig. 5:**
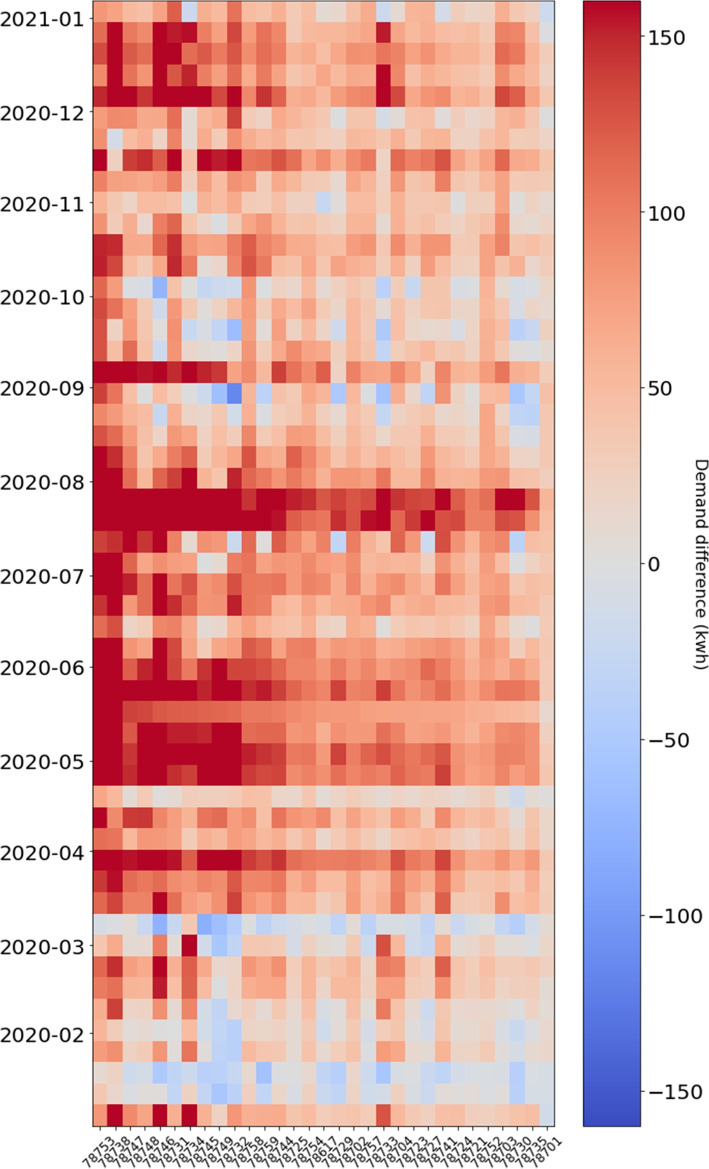
Weekly energy demand difference (kWh) for residential buildings by zip code

**Fig. 6:**
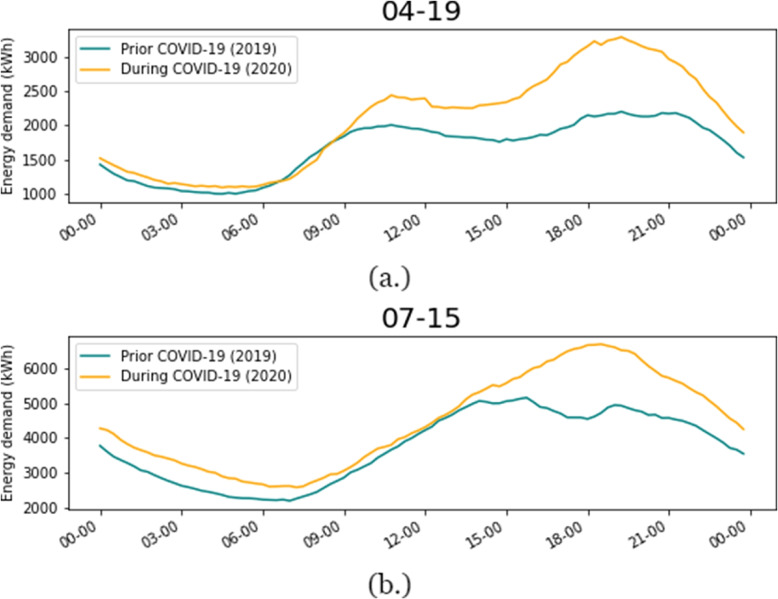
Raw energy consumption comparison for period (**a**) after SDPs (**b**) after Texas Open Phases

To go a step further, the heatmap of the energy demand difference for residential buildings at the zip code level which is aggregated on a weekly scale is presented in Fig. 5. We can see the two pinnacles in April and July that former one is caused by the SDPs, but for the latter, the energy usage is enlarged since the increased time of using air-conditioning because people spend more time working remotely during the summer time. Moreover, Fig. 6 illustrates the energy consumption patterns of the pinnacles in prior COVID and during COVID periods, which demonstrate the fluctuation of residents’ living habits. In Fig. 6 (a), people tend to awake later probably due to the absence of commuting time, and following larger energy consumption clearly indicates the change of demand by the SDPs. In the latter period after Texas Open Phases, Fig. 6 (b) shows the shift back in the morning while the overconsumption in the afternoon and night because of the extending policy of the SDPs which from company level instead of government level.

**Table 6:** Selected SVI variables for analysis COVID-19 impact

Variable Name	Description
E_DAYPOP	Estimated daytime population
MP_PCI	Per capita income estimate
EPL_UNEMP	Percentage of civilian (age 16+) unemployed estimate
F_THEME1	Sum of Socioeconomic Status variables
F_TOTAL	Sum of all the SVI variables

**Fig. 7:**
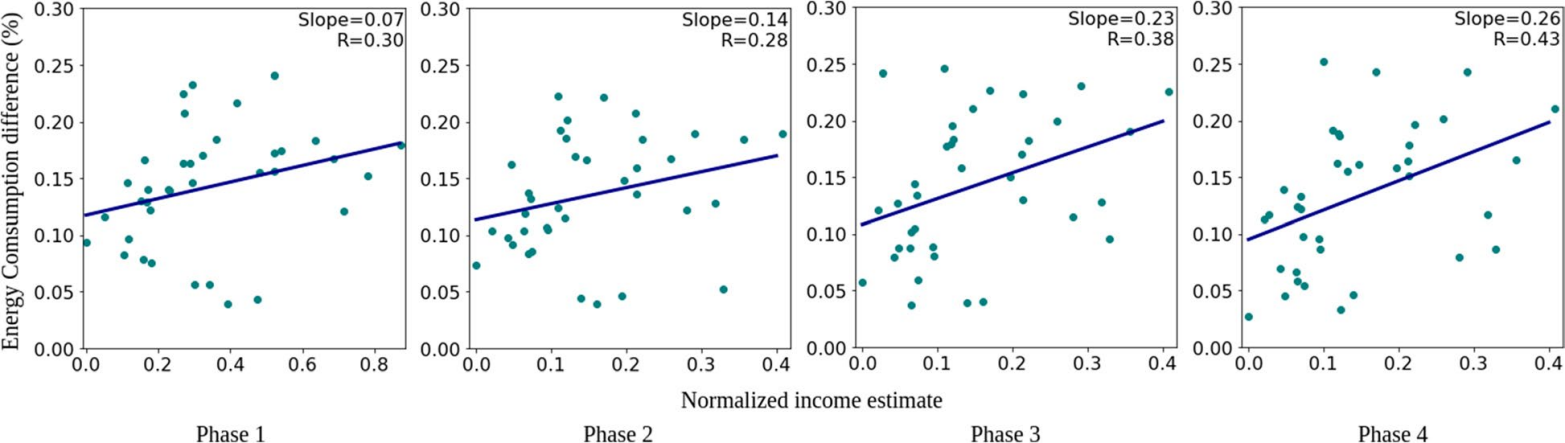
The scatter plot with linear regression between percentage energy consumption differences and normalized income estimation in each period

Besides looking for the spatial comparison, we also inspect the trend of how the SVI index and race distribution would interact with energy consumption differences through different phases during COVID. We applied the variables from the socioeconomic status domain in SVI, and five variables are selected as representatives while the code and detail descriptions are shown in Table 6. First, we conduct a linear regression model for each social variable with the calculated percentage of energy consumption difference through each period that we predefined in Table 1. Figure 7 demonstrates the distribution while the y-axis is the percentage energy consumption differences and the x-axis is the normalized value of SVI or race index. Every point represents one specific zip code area, and the regressed line illustrates whether the energy consumption tends to increase or decrease compared to synthesized data during that period. For instance, Fig. 7 shows that the estimated income level is consistently positively correlated with energy consumption difference, which implies the assumption that higher income contributes to higher usage of residential electricity. Moreover, the increased slopes in phases 3 and 4 suggest a dominant sensitivity of COVID cases to the energy consumption growth based on the state of affairs in phase 3 and 4.

**Fig. 8:**
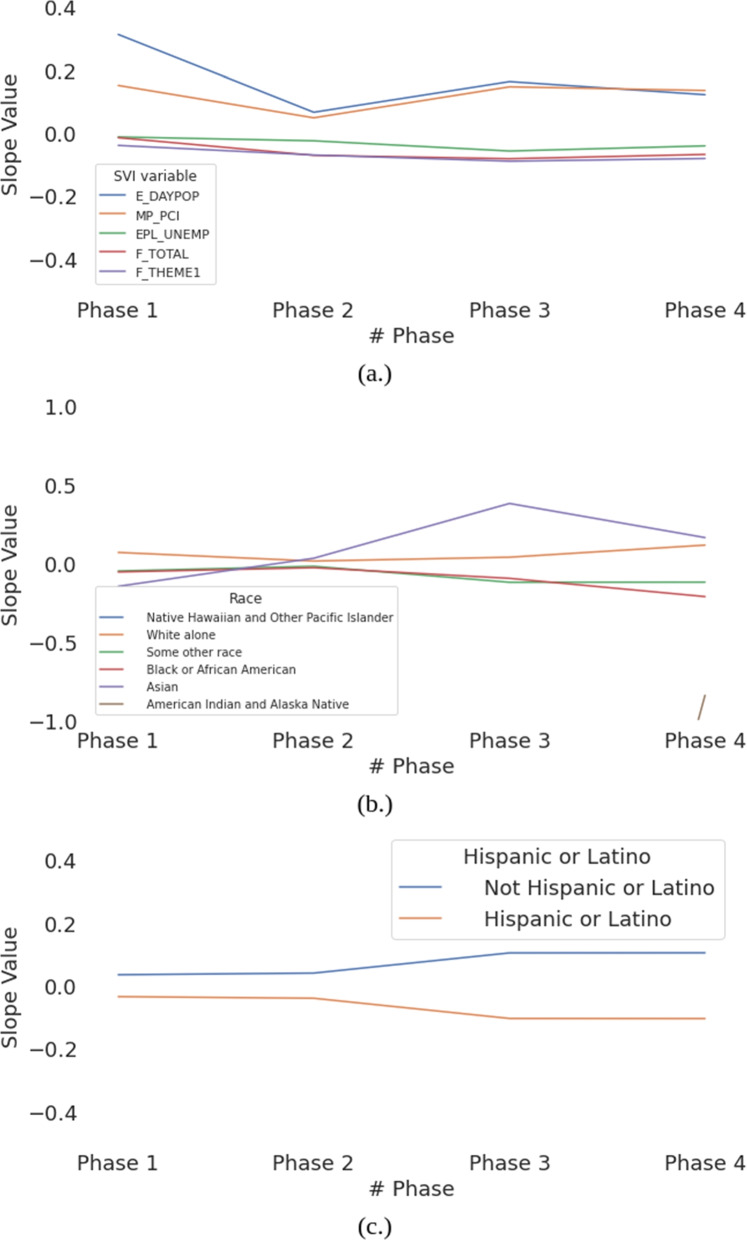
The slope value regressed with the percentage difference of energy consumption and (**a**.) SVI index, (**b**.) Single Race data, (**c**.) Hispanic

After concatenating the slope values of each period, Fig. 8 visualize the relationship between energy consumption differences and social factors. For the SVI indexes, Fig. 8 (a) first shows the dynamic of different SVI indexes to reflect the impact of COVID-19 in different stages. The overall SVI indexes (F_TOTAL and F_THEME1) hint the lower energy usage in all four phases while the daytime population and the income level suggest a tendency for lower energy consumption during the pandemic. Figure 8 (b) indicates the high variability among the single race in Austin. Compared to the inscrutable relationship among all races in phase 1 and 2, White alone and Asian have strong preferences for greater energy usage in phase 3 and 4. What can be clearly seen in Fig. 8 (c) is the distinction between Hispanic and non-Hispanic in phase 3 and 4. Those three figures not only indicate the general social causality in Austin due to the COVID impact but also illustrate the variety of energy poverty between different social factors.

## Discussion

Compared to most of the previous works that predict energy demand by Neural Networks, using tree-based methods are more preferred in this study. During the implementation of the ensemble model, most of the machine learning methods are fundamentally evaluated such as multivariate linear regression, support vector machine, and neural network. However, the results from those boosting-based models are either generating too smooth data or being practically inefficient that costs too much time to tune. Especially for those periodic data like energy consumption, air quality concentrations (Zimmerman et al., 2018), and temperature (Qasem et al., 2019), some researchers state the importance of tree-based ML methods. Although there are several studies that support using deep learning to build powerful neural networks with multiple layers, the attached overfitting phenomenon, and the time-consuming preparation and preprocessing of the data are inevitable. On the other hand, those traditional ML models formed by decision trees are more robust with enough numbers of estimators. Among all the methods in this research, XGBoost and LightGBM are more stable than AdaBoost and RF in the result from Section 4 because they involve both bagging and boosting concepts in the fitting process. Despite the fact that both methods do not achieve the best performance in the evaluation, they are more reliable due to the lower variation between different datasets. To conclude, picking samples from the ensemble tree is generally more efficient than calculating linear results from kernel fitting methods.

For the impact of COVID-19, most of the analysis results are associated with our expectations and preceding researches. The rising demands in residential buildings are clear. In the temporal dimension, the trend is highly related to the implementation of SDPs as well as the peak of COVID-19 cases. Commercial buildings in general are more adapted to the policies, so the corresponding changes to the residential ones do not occur to the commercial distribution of July shown in Fig. 3. However, due to the complexity of the commercial type, the impact is difficult to be quantified. Commercial electricity demands, in some way, are varied from industry to scale. For instance, the usage patterns of office buildings and of restaurants can be divergent, which cause the normalized data noisy and hard to estimate. Same circumstance happens in the spatial dimension. Highly concentrated commercial data in the north and central Austin create a dilemma of lacking information for other areas, and also the spatial counterfactual estimations indicate heterogeneous changes in zip code level. Still, a few areas in central Austin are following the offset relationship with the residential data, which are probably near the campus. Residential demands, in contrast, have homogeneous performances in the comparison between counterfactual estimations and observations. Underestimations of the residential energy consumption happen in almost all the areas, and the differences are more focusing on western Austin. In the context of that, higher income people, those who are mostly located in that area, are more adapted to the change by SDPs could be observed by comparing Fig. 4(f). Those who have room to spare are probably doing some non-physical job so that they could persist in their living without physical contacts. Further inspecting Fig. 4(e), it’s clear that the zip codes with the highest earnings and the longest work from home patterns are also the predominantly white neighborhoods, demonstrating the racially disparate impact of COVID-19 on the city.

Nonetheless, income level is not the only social factor that involved to affect energy consumption in Austin. The energy injustice could be implied by Figs. 7 and 8. Figure 7 reveals a consistency of positively correlated income level with energy consumption. This connection, on the one hand, supports that income level is crucial. However, on the other hand, this also suggests spatial diversity since there are not all the points are aligned with the line. Based on Fig. 8 (a), some SVI variables are inconsistent with the tendency of overusing energy. In fact, those indices imply a slight decline relationship with energy usage. This observation indicates the difficulty in determining the injustice community that not only lower-income people should be aware of but also other variables such as the dense daytime population area in this study. Furthermore, the community changes dynamically with time. Race data which in Fig. 8 (b) suggest that even the same community could have high fluctuation. Asian is a good illustration of the fluctuation that it starts on the negative side but ends on the positive side. That trend, somehow, supports the fact that the vulnerable community in energy consumption during COVID-19 is a dynamic process and could be determined by our proposed method.

It is also interesting to see the percentage difference generally increase in phase 3 and phase 4. As the state of affairs in phase 3 and phase 4 are essentially derived based on the sudden increase of COVID cases, it is surprising that the impact of the higher COVID case is greater than that of government policy. For example, E_DAYPOP from Fig. 8 (a) and Asian from Fig 8 (b) increase at the third stage, which implies that the Texas Open Phases strategy does not affect certain communities since their energy usage increased instead of returning back to pre-COVID levels. In Fig. 8 (c), we can observe that the non-Hinpanic group, after the increase in COVID cases in Phase 3, has the ability to stay at home while being productive.

That finding also leads to injustice issues in long-term disasters in Texas. The lockdown could protect people in short term, but how should the local government act to balance between social injustice and domestic economics? This study preliminary demonstrates a way to find it using energy consumption and social factors on a varied spatial and temporal scale. We also observe that certain people still use more energy even though the government had announced the opening policy. Having the awareness of the pandemic and the ability to adjust themselves to the social changes is critical, and this study demonstrates a way to examine the group without these kinds of abilities by the view of residential electricity.

## Conclusion

The study presents a novel framework for counterfactual modeling, and a thorough analysis of the long-term impact brought by COVID-19 on both commercial and residential energy demand. Most of the prior work that studied the impact was more focused on the discussion of the influence of COVID-19 using linear methods, which is insufficient to model the energy demand while the change by the pandemic. The counterfactual modeling method uses multiple powerful Machine Learning methods and stacking the predictions from them with a weighting scheme that has been dedicated compared. With precise and stable counterfactual explanations, our analysis quantifies the effect of COVID-19 in the Austin area using 13 million sub-hourly data from over 400,000 smart meters.

We confirm that both COVID-19 cases and government policies are highly related to the energy consumption data, and a permanent change to the patterns of electricity demand. Energy consumption during the COVID-19 pandemic is more affected by government policies at an early stage and has spatial variation while later with a greater impact from the actual COVID-19 cases. The impact was also investigated through the socioeconomic perspectives, hinting that higher income areas had bigger energy demand shifts due likely to more sustained working-from-home possibilities. We also conduct a detailed analysis of evaluating the social dynamic by comparing the difference between the observed and predicted energy consumption with multiple socioeconomic variables and race data, which provide a novel perspective to observe the impact of COVID-19 on residential energy consumption.

## Data Availability

The authors do not have permission to share the smart meter data.

## Code availability

The custom code built on Python and its public libraries can be made available upon reasonable request.
